# Biochemical and structural characterization of the apicoplast dihydrolipoamide dehydrogenase of *Plasmodium falciparum*

**DOI:** 10.1042/BSR20140150

**Published:** 2015-01-14

**Authors:** Larissa M. Laine, Marco Biddau, Olwyn Byron, Sylke Müller

**Affiliations:** *Institute of Infection, Immunity and Inflammation, College of Medical, Veterinary and Life Sciences, University of Glasgow, Glasgow G12 8TA, U.K.; †School of Life Sciences, College of Medical, Veterinary and Life Sciences, University of Glasgow, Glasgow G12 8TA, U.K.

**Keywords:** pyruvate dehydrogenase complex, apicoplast, redox homoeostasis, recombinant expression, gene deletion, protein structure, 1-CysPx, 1-Cys-peroxredoxin, 2-CysPx, 2-Cys-peroxredoxin, 5-FC, 5-fluorocytosine, AUC, analytical ultracentrifugation, BSO, L-buthionine sulfoximine, DAM, dummy atom model, DHLA, dihydrolipoamide, DMD, discrete molecular dynamics, E1, pyruvate decarboxylase, E1α, α subunit of E1, E2, dihydrolipoamide acetyltransferase, E3, dihydrolipoamide dehydrogenase, E3BP, E3-binding protein, GST, glutathione S-transferase, *hdhfr*, human dihydrofolate reductase, HRP, horseradish peroxidase, KADH, α-keto acid dehydrogenases, mE3, mitochondrial E3, ORF, open reading frame, PDC, pyruvate dehydrogenase complex, *Pf*aE3, *Plasmodium falciparum* aE3, SAXS, small-angle X-ray scattering, SBD, sub-unit binding domain, SE, sedimentation equilibrium, SV, sedimentation velocity, TCA, tricarboxylic acid

## Abstract

PDC (pyruvate dehydrogenase complex) is a multi-enzyme complex comprising an E1 (pyruvate decarboxylase), an E2 (dihydrolipomide acetyltransferase) and an E3 (dihydrolipoamide dehydrogenase). PDC catalyses the decarboxylation of pyruvate and forms acetyl-CoA and NADH. In the human malaria parasite *Plasmodium falciparum*, the single PDC is located exclusively in the apicoplast. *Plasmodium* PDC is essential for parasite survival in the mosquito vector and for late liver stage development in the human host, suggesting its suitability as a target for intervention strategies against malaria. Here, *Pf*aE3 (*P. falciparum* apicoplast E3) was recombinantly expressed and characterized. Biochemical parameters were comparable with those determined for E3 from other organisms. A homology model for *Pf*aE3 reveals an extra anti-parallel β-strand at the position where human E3BP (E3-binding protein) interacts with E3; a parasite-specific feature that may be exploitable for drug discovery against PDC. To assess the biological role of *Pfae3*, it was deleted from *P. falciparum* and although the mutants are viable, they displayed a highly synchronous growth phenotype during intra-erythrocytic development. The mutants also showed changes in the expression of some mitochondrial and antioxidant proteins suggesting that deletion of *Pfae3* impacts on the parasite's metabolic function with downstream effects on the parasite's redox homoeostasis and cell cycle.

## Abstract 2

The malaria parasite dihydrolipoamide dehydrogenase is active as a dimer and has specific structural features which could be exploitable for drug discovery. The enzyme is not essential for blood stage development but loss of function affects redox homoeostasis and cell cycle.

## INTRODUCTION

The PDC (pyruvate dehydrogenase complex) belongs to the KADH (α-keto acid dehydrogenases), a family of mega-Dalton multi-enzyme complexes comprising multiple subunits of three different enzymes. In mammalian, yeast and nematode PDC, the E2 (dihydrolipoamide acetyltransferase) together with the E3BP (E3-binding protein) form the complex core [1–5], whereas in plants and bacteria it is E2 alone that generates the core of the PDC complex [6–8]. This core structure forms either a dodecahedral 60-mer, as found in PDC from humans, Gram-positive bacteria and plants, or it generates an octahedral 24-mer as is found in Gram-negative bacteria [7,9–11]. PDC E2 catalyses the transfer of the acetyl group from S-acetyldihydrolipoamide, a covalently attached co-factor of E2, to CoA producing acetyl-CoA. The E1 (pyruvate decarboxylase subunit) of eukaryotes and Gram-positive bacteria is a heterotetramer composed of two subunits, E1α (α subunit of E1) and E1β (β subunit of E1), whereas in Gram-negative bacteria, the enzyme is a homodimer [12]. PDC E1 transfers the acetyl group from pyruvate to the thiamine pyrophosphate co-factor, concurrently releasing CO_2_. The acetyl moiety is transferred from PDC E1 to the lipoamide co-factor of E2 which transfers it to CoA to form acetyl-CoA. During this reaction the lipoamide co-factor is reduced to DHLA (dihydrolipoamide) and, in order to allow catalysis to proceed, E3 (dihydrolipoamide dehydrogenase) re-oxidizes the co-factor, generating NADH. Both E1 and E3 bind to the E2 core to allow substrate channelling, which is facilitated by the so-called ‘swinging arm’, referring to the lipoamide co-factor that is covalently attached to the lipoyl-domains of PDC E2 [13].

In eukaryotes, PDC is located in the mitochondria, linking cytosolic glycolysis to the mitochondrial TCA (tricarboxylic acid) cycle; plants possess mitochondrion- and chloroplast-located PDCs and the plastid-located PDC is vital for providing acetyl-CoA for fatty acid biosynthesis exclusively occurring in the organelle [14]. *Plasmodium* possesses a single PDC that is found solely in the apicoplast [15], a plastid-like organelle found in most apicomplexan parasites, where it provides acetyl-CoA for fatty acid biosynthesis, similar to plant chloroplast PDC [16,17]. It was found that PDC is essential during late liver stage development in mouse malaria species [16], whereas in *Plasmodium falciparum*, one of the human infective malaria species, recent studies suggest that PDC and also fatty acid biosynthesis activity are important for the development of infective sporozoites at the end of sexual development in the *Anopheles* vector [18,19]. The multi-enzyme structure of PDC does however make it probable that the loss of one protein will not necessarily interfere with the function(s) of all protein members of the enzyme complex.

Therefore we studied the effects on *P. falciparum* of the deletion of *Pfae3* (*Plasmodium falciparum* aE3) (PF3D7_0815900), encoding dihydrolipoamide dehydrogenase, an enzyme that is an essential component of apicoplast PDC. The enzyme may also have an impact on parasite's redox homoeostasis in its own right. With the aim of informing future anti-malarial drug discovery against exo-erythrocytic parasite stages, *Pf*aE3 was also recombinantly expressed and its biochemical and biophysical characteristics determined.

## EXPERIMENTAL

### Cloning, recombinant protein expression and purification of *Pfa*E3

Mature-length *Pf*aE3 (comprising amino acids 118–667) was cloned into the vector pQE30 (Qiagen) using genomic *P. falciparum* 3D7 DNA as a template and the forward primer 5′-GCGCGGATCCTTAAAAGGAAGTACAC-3′ (starting at nucleotide 331 to remove the N-terminal apicoplast-targeting peptide) and the reverse primer 5′GCGCAAGCTTTTAGTGAGTTCTTATTTTTGATATAG-3′ containing a BamHI and a HindIII restriction site, respectively, to allow directional cloning into the pQE30 expression vector. Recombinant mature length *Pf*aE3 carrying an N-terminal His-tag was expressed overnight at 30°C in NovaBlue (DE3) *Escherichia coli* following induction with 0.5 mM IPTG (isopropyl β-D-1-thiogalactopyranoside). Bacteria were harvested by centrifugation at 3000 ***g*** for 15 min at 4°C and the pellets were resuspended in lysis buffer (50 mM sodium phosphate, 300 mM NaCl, 10 mM imidazole, pH 8.0) containing protease inhibitors (20 μM leupeptin, 2 μM pepstatin A, 1 mM PMSF, 1 mM benzamidine, 2 μM 1,10-phenanthroline and 10 μM E-64) and 100 μM flavin adenine dinucleotide. Resuspended bacterial pellets were incubated on ice for 30 min with 50 μg/ml lysozyme (Sigma), 1 μg/ml DNase (Roche) before they were disrupted using a OneShot Cell Disrupter (Constant Systems) and the lysate was centrifuged at 48000 ***g*** for 1 h. The resulting supernatant was filtered through a 45 μm Millex-HV PVDF syringe filter (Millipore) before applying it to Ni-NTA (Ni^2+^-nitrilotriacetic acid) agarose (Qiagen) and incubating for 1 h at 4°C with rotation on a blood wheel. The mix was then poured into an Econo-Pac chromatography column (Bio-Rad) and the flow-through was collected by gravity flow. The Ni-NTA resin was washed twice with 4 volumes of wash buffer 1 (50 mM sodium phosphate, 300 mM NaCl, 20 mM imidazole, pH 8.0) and subsequently with 1 volume wash buffer 2 (50 mM sodium phosphate, 300 mM NaCl, 50 mM imidazole, pH 8.0), before the recombinant protein was eluted with 50 mM sodium phosphate, 300 mM NaCl, 250 mM imidazole, pH 8.0. Protein purity was assessed by SDS–PAGE (10% gel). Fractions containing the recombinant protein were pooled and applied to a previously calibrated HiLoad 16/60 Superdex 200 chromatography column for further purification. The column was equilibrated with 50 mM potassium phosphate, 150 mM NaCl, 2 mM EDTA, pH 7.4. The calibration standards used were dextran blue (2 MDa, 0.6 mg/ml), apoferritin (440 kDa, 2.5 mg/ml), alcohol dehydrogenase (150 kDa, 2.5 mg/ml), bovine serum albumin (66 kDa, 2.5 mg/ml), carbonic anhydrase (29 kDa, 2.5 mg/ml) and cytochrome *c* (12 kDa, 2.5 mg/ml). The elution fractions containing *Pf*aE3 were analysed by SDS–PAGE (10%) and Western blotting.

### Activity assays

The catalytic activity of *Pf*aE3 was assessed as described by McMillan et al. [20]. The generation or consumption of NADH was determined spectrophotometrically by measuring the change in absorbance at 340 nm in a UV-2501 spectrophotometer (Shimadzu). The extinction coefficient of NADH at 340 nm (6220/M/cm) was used to calculate the specific activity of recombinant *Pf*aE3 in the forward and reverse reactions. One unit of *Pf*aE3 in the forward reaction activity is defined as 1 μmol of NAD^+^ reduced per min per mg of enzyme, while 1 unit of *Pf*aE3 activity in the reverse reaction is defined as 1 μmol of NADH oxidized per min per mg enzyme. The forward reaction was performed in 50 mM potassium phosphate, 1 mM EDTA, pH 8 with 1 μg of recombinant *Pf*aE3 at 25°C. A 60 mM stock of DHLA (Sigma) was freshly prepared in 100% (v/v) ethanol and 10 mM NAD^+^ was freshly prepared with reaction buffer. The *K*_m_ and *v*_max_ values for DHLA were determined by keeping NAD^+^ constant at 2 mM and varying the concentration of DHLA (50 μM–2 mM). The kinetic parameters for NAD^+^ were determined, keeping the DHLA concentration constant at 2 mM and varying the NAD^+^ concentration (62.5 μM and 2 mM). The reverse reaction was performed in 50 mM potassium phosphate, 1 mM EDTA, pH 7, at 25°C with 1 μg of *Pf*aE3. Lipoamide (Sigma) was freshly prepared with 100% ethanol as a 60 mM stock solution. NADH was prepared in reaction buffer as a 10 mM stock solution. To determine the *K*_m_ and *v*_max_ values for lipoamide, the concentration of NADH was kept constant at 200 μM and lipoamide was varied between 50 μM and 4 mM. For the kinetic parameters for NADH, lipoamide was kept constant at 2 mM and NADH was varied between 10 and 200 μM.

### Analytical ultracentrifugation (AUC)

AUC analysis was performed using a Beckman Coulter Optima XL-I analytical ultracentrifuge (Palo Alto). SV (sedimentation velocity) experiments were carried out at 49000 rpm at 4°C. Equal (360 μl) volumes of protein (ranging from 3.1 to 22.7 μM) and reference solvent (50 mM potassium phosphate, 250 mM NaCl, 2 mM EDTA, pH 7.4) were loaded into 12 mm charcoal-filled epon double sector centerpieces. Concentration distributions (200) were recorded every 2 min using absorbance optics. The program SEDFIT [21,22] was then used to model the SV profiles with finite-element solutions of the Lamm equation for a large number of discrete, non-interacting species resulting in a continuous size distribution [c(s) versus s]. Initial fits were conducted over the range of 0.0–25 S to cover all plausible species. High-resolution (resolution=200) fits were then performed within a narrower range (0–15 S, which transformed to 0.0–26.1 S when standardized to the 20°C, water scale). Using SEDNTERP [23] (http://sednterp.unh.edu/) the partial specific volume of *Pf*aE3 (0.732 ml/g at 4°C and 0.739 ml/g at 20°C) and the reference buffer density and viscosity (ρ=1.01804 g/ml and η=1.6354 cPoise, respectively, at 4°C) were computed. The sedimentation coefficients obtained by integration of the c(s) peaks were plotted against protein concentration to obtain the sedimentation coefficient at infinite dilution (*s*^0^_20, *w*_) from the y-intercept.

SE (sedimentation equilibrium) experiments were carried out at 17000 rpm at 4°C. Equal volumes (80 μl) of *Pf*aE3 at concentrations ranging from 4.7 to 22.7 μM and reference buffer (50 mM potassium phosphate, 250 mM NaCl, 2 mM EDTA, pH 7.4) were loaded into double sector 12 mm path length centrepieces. As interference optics were used to record data, the laser delay, fringe contrast and brightness at the experimental speed were adjusted before the run to obtain high-quality fringes. Ten scans were recorded over a radial range of 6.80–7.25 cm and were taken 3 h apart following a 3 h initial delay. WinMATCH (Jeffrey Lary, University of Connecticut, Storrs, CT, USA) was used to confirm that equilibrium had been reached in the sample. SE data were fit using the species analysis model in SEDPHAT [24,25] (http://www.analyticalultracentrifugation.com/sedphat/sedphat.htm).

### Small-angle X-ray scattering

SAXS (small-angle X-ray scattering) data were acquired on the EMBL beamline X33 at DESY, Hamburg. The beam current was 100–140 mA and the X-ray wavelength was 1.5 Å. The sample to detector distance was 2.7 m giving a momentum transfer (*s*=4π sin (θ)/λ, where 2θ is the scattering angle and λ is the X-ray wavelength) range of 0.006≤*s*≤0.6 Å^−1^. Data were collected with a 2D photon counting Pilatus 1M-W pixel X-ray detector, which was calibrated with a standard sample of 5 mg/ml bovine serum albumin. *Pf*aE3 was purified two days before measurements and homogeneity was determined by SV analysis before and after measurements. Protein activity was also confirmed on the day of purification. SAXS data were acquired at 10°C for *Pf*aE3 at concentrations of 9, 8, 6, 5 and 4 μM. The exposure time of the sample to the X-rays was 15 s and eight frames were taken for each sample. The raw data were processed with an automated SAXS data processing pipeline [26] for initial averaging of sample data frames and buffer subtractions. The ATSAS 2.5 programme suite [27] (http://www.embl-hamburg.de/biosaxs/software.html) was used for all subsequent processing and analysis of SAXS data, apart from ScÅtter (http://www.bioisis.net/tutorial/9) which was used to generate dimensionless Kratky plots. PRIMUS [28] was used to assess the data for aggregation and to perform Guinier analysis. High-angle data for the 9 μM protein and low-angle data for the 4 μM protein were merged and the resulting scattering curve was used for all subsequent analyses, including determination of the *p*(*r*) distance distribution function and the maximum dimension (*D*_max_) using GNOM [29]. *R_g_* was determined both from the Guinier approximation and the *p*(*r*) distribution.

### Structural modelling

*Ab initio* modelling of the solution conformation of *Pf*aE3 from the SAXS data using P2 symmetry was carried out using DAMMIF [30] on the ATSAS online server (http://www.embl-hamburg.de/biosaxs/atsas-online/dammif.php) [26]. An atomic resolution model for a monomer of *Pf*aE3 was constructed from a combination of models generated using the PHYRE2 server [31] and the I-TASSER server [32]. This was then superimposed on one chain of the dimer structure of human E3 (PDB ID: 2F5Z, [33]) using PyMol (Schrödinger, LLC) and the process repeated for the second chain of human E3 in order to gain an overview of how a putative *Pf*aE3 dimer would compare with its human counterpart.

### *P. falciparum* culture

*P. falciparum* 3D7 (The Netherlands) was cultured according to Trager and Jensen [34] in RPMI 1640 (Invitrogen) containing 11 mM glucose, 0.5% (w/v) Albumax II (Invitrogen), 200 μM hypoxanthine, 20 μg/ml gentamycin (PAA) in human erythrocytes between 0.5 and 5% (w/v) haematocrit. Parasite cultures were maintained under an atmosphere of reduced oxygen [1% (v/v) oxygen, 3% (v/v) CO_2_ and 96% (v/v) nitrogen]. Parasites were synchronized using sorbitol [35] and freed from erythrocytes using saponin [36]. Parasitaemia was determined using Giemsa-stained thin smears.

### Generation of Pfae3 knockout construct and transfection of P. falciparum

The 5′ and 3′ ends of *Pfae3* were cloned into pCC1 [37] flanking the *hdhfr* (human dihydrofolate reductase) selectable marker cassette. The primers used to amplify the 5′ fragment [nucleotides 37–526 of the *ae3* ORF (open reading frame)] were *ae3-5*′s: 5′-GAGCACTAGTCTTAACGTCGTTACTCTAATTTGGTATC-3′ and *ae3-5*′as: 5′-GAGCCTTAAGGCGCTTTGCTTGG-TATACAGCC-3′ containing an SpeI and AflII restriction site, respectively. The primers used to amplify the 3′ fragment (nucleotides 1456–2001 of the *ae3* ORF) were *ae3-3*′s: 5′-GAGCGAATTCGCACACACAGCATCATATCAAG-3′ and *ae3-3*′as: 5′-GAGCCCTAGGTTAGTGAGTTCTTATTT-TTGATATAGA-3′ containing an EcoRI and AvrII restriction site, respectively. The nucleotide sequence of pCC1-Δ*Pfae3* was verified (Eurofins MWG Operon).

Transfection of pCC1-Δ*Pfae3 P. falciparum* 3D7 erythrocytic stages was performed as described previously [38]. Transfectants were selected with 2.5 nM WR99210. Before cloning by limiting dilution according to Kirkman et al. [39], pCC1-Δ*Pfae3* transfectants were subjected to negative selection with 1 μM 5-FC.

### DNA and protein extraction

Parasites were freed from erythrocytes using saponin lysis and genomic DNA was isolated by resuspending the washed pellet in 50 mM Tris–HCl, pH 9 containing 0.2 M NaCl, 0.1 M EDTA, 1 mg/ml proteinase K and 1% (w/v) SDS and incubated overnight at 37°C with rotation. This was followed by phenol extraction and the genomic DNA was precipitated with isopropanol. After washing the genomic DNA with 70% ethanol it was dissolved in 200 μl of Tris–HCl, pH 8.0 containing 1 mM EDTA.

For protein extraction the parasite pellet was resuspended in 2D-lysis buffer (100 mM Hepes pH 7.4, 5 mM MgCl_2_, 10 mM EDTA, 0.5% (v/v) Triton X-100, 5 μg/ml RNase A, 1 mM PMSF, 1 mM benzamidine, 2 μg/ml leupeptin, 10 μM E-64, 2 mM 1,10- phenanthroline, 4 μM pepstatin A). The parasite pellet was freeze-thawed three times in dry ice and sonicated for 5 min in a sonicating water bath at 4°C. Subsequently the samples were centrifuged at 13000 ***g*** at 4°C for 15 min and the protein concentration of the supernatant was determined using the Bradford method with bovine serum albumin as a standard [40].

### Western blotting

Separation of proteins (20 μg) was performed by SDS–PAGE using NuPage Novex 4–12% and 10% (w/v) bis-Tris gels (Invitrogen). Proteins were either stained with Coomassie Brilliant Blue or were transferred to Protran nitrocellulose membranes (Schleicher & Schuell) using a Transblot semi-dry transfer system (BioRad). Western blots were blocked in 5% (w/v) non-fat dried skimmed milk dissolved in PBS overnight at 4°C before the primary antibodies were applied for 1 h at room temperature. For protein identification, a mouse anti-His-tag antibody (BD Biosciences) was used at 1:25000 dilution; after three washes the membranes were then incubated with HRP (horseradish peroxidase) conjugated secondary antibody (1:10000, anti-mouse-HRP; Promega) for 1 h at room temperature before the blot was washed three times and developed using the Immobilon Western Chemiluminescent substrate (Millipore) following manufacturer's instructions. The signals were visualized by exposing the blots to X-ray films.

For relative quantification analyses, the blots were probed simultaneously with *P. falciparum* rabbit anti-actin antibody (1:12000, used as loading control) and one antibody of interest (*P. falciparum* rabbit anti-branched chain α-keto acid dehydrogenase E2 antibody at 1:5000; *P. falciparum* rabbit anti-isocitrate dehydrogenase antibody at 1:10000; *P. falciparum* rabbit anti-mitochondrial E3 antibody at 1:2500; *P. falciparum* rabbit anti-malate dehydrogenase at 1:2000; *P. falciparum* rabbit anti-glutathione reductase antibody at 1:2500; *P. falciparum* rabbit anti-GST (glutathione S-transferase) antibody at 1:2500; *P. falciparum* rabbit anti-1-CysPx (1-Cys peroxiredoxin) antibody 1:50000; *P. falciparum* rabbit anti-2-CysPx (2-Cys peroxiredoxin) antibody at 1:70000; *P. falciparum* rabbit anti-PDC—E2 lipoyl—domain antibody at 1:250). The membranes were then washed three times in PBS and probed for 1 h at room temperature with an IR dye-conjugated antibody (1:10000, IRDye 800CW goat anti-rabbit antibody; LI-COR biosciences). After three PBS washes the fluorescent signals were acquired with the Odyssey SA scanner (LI-COR biosciences) and band intensities quantified with the provided software.

### Southern blotting

Two to 3 μg of genomic DNA and 0.2 ng of the pCC1-Δ*Pfae3* plasmid were digested at 37°C overnight with HincII. The digested DNA was separated on a 0.8% (w/v) agarose gel and subsequently blotted onto Hybond N^+^ membranes. The membranes were probed with the *PfaE3* 5′ fragment cloned into pCC1 (nucleotides 37–526 of the *ae3* ORF), which was labelled using the Gene Images AlkPhos Direct Labelling kit (GE Healthcare) according to the manufacturer's recommendations. The membranes were probed overnight at 60°C and then washed as recommended by the manufacturer. Visualization of DNA fragments on the membranes was achieved using CDP Star detection solution (GE Healthcare) followed by exposure of the membrane to autoradiography film.

### Growth assay

3D7^∆*Pfae3*^ growth was determined as described by Günther et al. [41] with modifications. Cultures were synchronized twice during 4 h. The ring stage parasites were diluted to 0.5% parasitaemia and 5% haematocrit in 2 ml of RPMI complete medium. Each parasite line was analysed in triplicate. Giemsa stained thin smears were prepared daily and the cultures were diluted 1:5 with fresh erythrocytes every second day. The parasitaemia was determined by counting 1000 erythrocytes. In addition, the development of the erythrocytic stages was monitored by counting 100 parasites per slide distinguishing rings, trophozoites and schizonts.

### Determination of IC_50_ values

The incorporation of [^3^H]-hypoxanthine was used to determine IC_50_ values [42] for BSO (L-buthionine sulfoximine), paraquat and triclosan. The starting concentrations for the agents were 1.25 mM for BSO, 0.5 mM for paraquat and 0.4 mM for tricolsan. Following incubation for 48 h, the medium (without further addition of drugs) was replaced and 5 μCi [^3^H]-hypoxanthine per ml was added to each well. The plates were incubated for a further 24 h after which they were frozen at −20°C. The plates were defrosted at room temperature for 2–3 h before harvesting with a Harvester 96™ Mach III (TomTec) onto Printed Filter Mat A filter mats (Perkin Elmer). These were dried at 55°C for 90 min and sealed into plastic sample bags after addition of 4 ml of scintillation fluid and determining incorporation of [^3^H]-hypoxanthine using a Wallac 1450 MicroBeta Trilux liquid scintillation counter (Perkin Elmer) for 1 min per well. IC_50_ values were calculated using GraphPad Prism 5.0.

## RESULTS

### Recombinant expression, purification and catalytic parameters of *Pf*aE3

**Figure 1:**
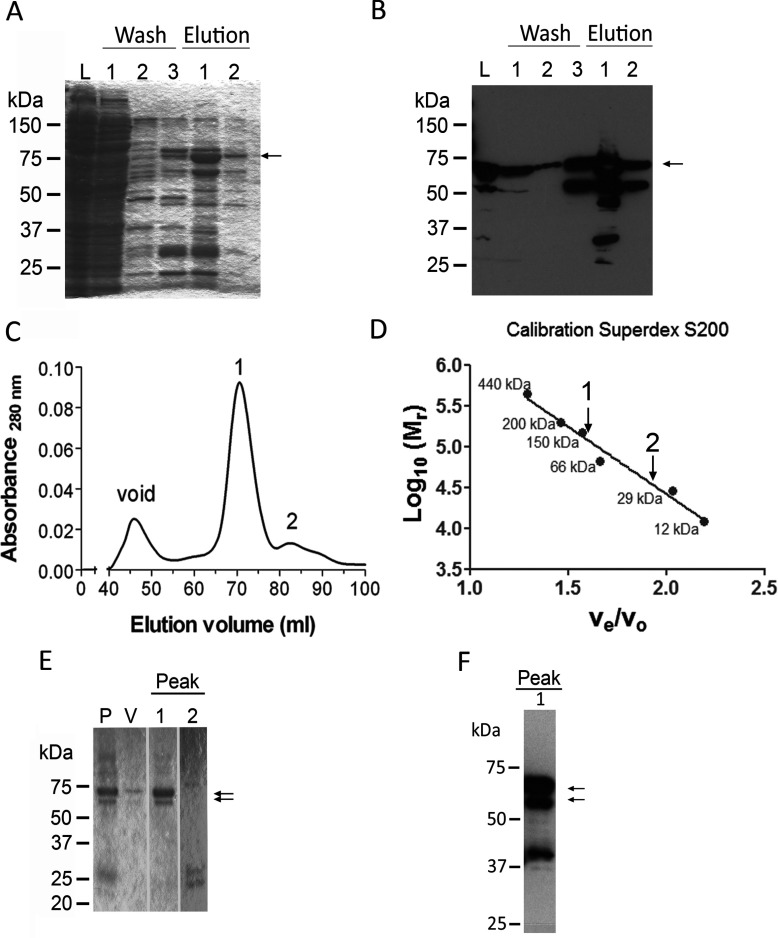
Purification of recombinant *Pf*aE3 (**A**) SDS**–**PAGE (10% gel) of recombinant *Pf*aE3 after Ni^2+^-NTA batch purification. Roughly 30–50 μg of protein were loaded per lane (lane L is the bacterial lysate applied to the resin and lane 1 of the wash fractions–50 μg each; lanes 2 and 3 of the wash fractions and lanes 1 and 2 of the elution fractions–30 μg each) and proteins were visualized with Coomassie Brilliant Blue. A protein of about 64 kDa (arrow) was enriched. (**B**) The enriched protein (arrow) was His-tagged as verified by Western blotting using an anti-His-tag antibody. Other lower molecular mass proteins also reacted with the antibodies; these are presumably degradation products. (**C**) The elution fractions 1 and 2 from the Ni^2+^-NTA batch purification containing *Pf*aE3 were applied to a HiLoad 16/60 Superdex S200 column previously calibrated with dextran blue (2000 kDa, void volume), apoferritin (440 kDa), β-amylase (200 kDa), alcohol dehydrogenase (150 kDa), BSA (66 kDa), carbonic anhydrase (29 kDa) and cytochrome *c* (12 kDa). The figure shows the elution profile featuring two major protein peaks (1 and 2). A third peak eluting at about 45 ml corresponds to the void volume of the column (void) and may be attributable to protein aggregates forming during the purification procedure. (**D**) Calibration curve of Superdex S200 column. Interpolation of the *v*_e_/*v*_0_ of *Pf*aE3 peak 1 with the calibration curve of the Superdex S200 column suggests that this protein has a molecular mass of about 140 kDa. Peak 2 represents a protein much smaller than *Pf*aE3 (~35 kDa). (**E**) SDS**–**PAGE (10% gel) shows that peak 1 from the gel filtration contained two closely migrating proteins of approximately 60–64 kDa (arrows). Peak 2 does not contain proteins of the expected size of *Pf*aE3. P, pooled Ni^2+^-NTA elutions applied to Superdex S200; V, protein eluted at void volume; 1 and 2, proteins eluted in peaks 1 and 2, respectively. (**F**) The protein present in peak 1 was confirmed to be *Pf*aE3 by western blotting with anti-His-tag antibody (arrows).

*P. falciparum* aE3 (*Pf*aE3) was recombinantly expressed in *E. coli* using an N-terminally truncated construct (amino acids 110–667) containing an N-terminal 6-His-tag. The protein was purified in a two-step process using Ni-NTA agarose batch purification followed by gel filtration on Superdex S200. The presence of the His-tagged protein (~64 kDa after Ni-NTA chromatography) was verified by Western blotting where substantive degradation of the recombinant protein was detected (Figure 1). Gel filtration resulted in the separation of the applied proteins into two major peaks (peaks 1 and 2) corresponding to proteins of ~140 and ~35 kDa (Figures 1C and 1D). SDS–PAGE revealed that peak 1 contained two polypeptides of approximately 60–64 kDa (Figure 1E). Both were His-tagged, as verified by Western blotting (Figure 1F) and presumably corresponded to full-length and a C-terminally truncated form of *Pf*aE3. As opposed to the previous expression trial reported by McMillan et al. [20], which obtained only marginal amounts of recombinant *Pf*aE3, the yield of recombinant *Pf*aE3 using the pQE30 expression plasmid was 1.5 mg/l of bacterial culture, which allowed for kinetic and structural analyses of the recombinant protein.

*Pf*aE3 was found to be catalytically active in both forward and reverse reactions (Table 1). In the forward reaction, the *K*_m_ for DHLA was about 10-fold higher than that of *P. falciparum* mE3 and the protozoan parasite *Trypanosoma cruzi* E3 [43] and compares well with the value for human E3 [20,43,44]. This is as opposed to the *K*_m_ determined for NAD^+^, which is five times lower than that of the E3s from other organisms. However, the turnover numbers of *Pf*aE3 for both substrates in the forward reaction are similar to those determined for E3 from other species, suggesting that at substrate saturation *Pfa*E3 is as catalytically competent as the mE3 (mitochondrial E3) proteins. This is not the same for the reverse reaction, where the k_cat_ for both NADH and lipoamide are well below those determined for *Pf*mE3 but compare favourably with the kinetic parameters determined for the *T. cruzi* protein [43].

**Table 1:** Kinetic parameters of *Pf*aE3 Forward and reverse reactions were followed and catalytic parameters determined as outlined in the Experimental Procedures. *Pf*aE3 values represent means ±S.E.M. of three independent measurements.

	*Pfa*E3	*Pfm*E3 [20]	*Homo sapiens* E3 [44]	*Trypanosoma cruzi* E3 [43]
Forward				
K_m_ DHLA (μM)	1160±105	146±15	570	130
k_cat_ DHLA (s^−1^)	110	135	382	166 (pH 7.0) [20]
				244 (pH 7.5)*
K_m_ NAD^+^ (μM)	96±12	450±30	290	600
k_cat_ NAD^+^ (s^−1^)	110	135 to 337	382	166 (pH 7.0) [20]
				244 (pH 7.5)*
Reverse				
K_m_ lipoamide (μM)	841±97	870±270	1010	800
k_cat_ lipoamide (s^−1^)	97	448	167	106 (pH 7.0) [20]
				91 (pH 7.5) [20]
K_m_ NADH (μM)	15±2	21±6	51	25
k_cat_ NADH (s^−1^)	77.8	448	167	106 (pH 7.0) [20]
				91 (pH 7.5) [20]

*Recalculated from the indicated reference assuming a molecular mass of 50 kDa for *T. cruzi* E3.

### *Pf*aE3 is a dimer

**Figure 2:**
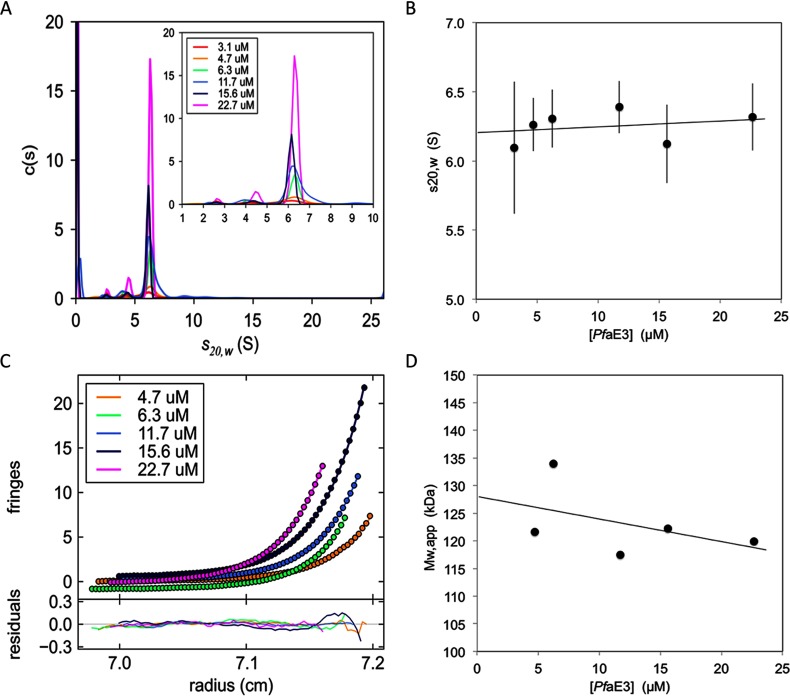
AUC analyses of *Pf*aE3 (**A**) Size distribution [c(*s*) versus *s*] analysis of AUC SV data with SEDFIT [21,22] was carried out for *Pf*aE3 at dimer concentrations from 3.1 to 22.7 μM. The main species accounted for 68–94% of the sample. Inset shows a ‘zoom’ into the analysis over the range 1.0≤*s*_20,*w*_≤10.0 S. (**B**) Concentration dependence of *s*_20,*w*_ from which the sedimentation coefficient at infinite dilution (*s*^0^_20, *w*_=6.2±0.1 S) was determined. The error bars represent the standard deviation shown as the square root of the central moment of the curve. (**C**) SE interference data (for clarity, only every 4th data point is shown for each concentration) were analysed with SEDPHAT [25] using a single species model for which the fit is shown (smooth line). Residuals of the individual fits are shown beneath the fitted data. (**D**) The molecular mass resulting from the fits in (**C**) are plotted as a function of *Pf*aE3 dimer concentration. At infinite dilution the molecular mass is 128 kDa. Plots in panels (**A**) and (**C**) were prepared using the program GUSSI (http://biophysics.swmed.edu/MBR/software.html).

In common with E3 from other organisms, *Pf*aE3 elutes in size exclusion chromatography as a homo-dimer of ~140 kDa (Figures 1C and 1D). This observation was verified by AUC. SV showed a main species (Figure 2A) with a sedimentation coefficient *s*^0^_20, *w*_ of 6.2±0.1 S (Figure 2B), comparable with that determined for the human E3 dimer (5.9 S, [45]) suggesting that the overall solution conformation and oligomerization state might be similar.

To determine the molecular mass of *Pf*aE3, SE analysis was undertaken. The data were fitted with a single species model (Figure 2C) and the resultant weight average molecular masses were plotted as a function of *Pf*aE3 dimer concentration (Figure 2D) yielding an infinite dilution whole-cell weight average molecular mass of 128 kDa, remarkably close to the molecular mass of the *Pf*aE3 dimer calculated from the amino acid sequence of the recombinantly expressed full-length protein (127478 kDa) (see above).

**Figure 3:**
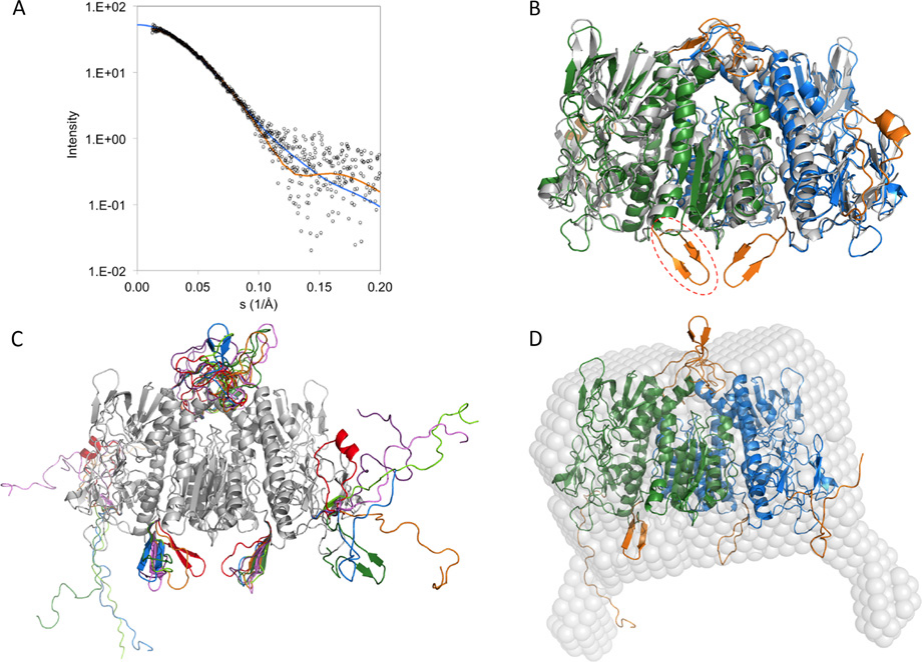
Modelling of *Pf*aE3 (**A**) SAXS data (open circles) with fits by (blue line, **φ**=0.823) the DAM shown in (**D**); (orange line, φ=0.787) the representative fleximer model after DMD optimization [in blue, green and orange in (**D**)]. (**B**) An atomic resolution model for a dimer of *Pf*aE3 constructed from a combination of models generated using the PHYRE2 server [31] and the I-TASSER server [32] superimposed on the dimer structure of human E3 (PDB ID: 2F5Z, [33]). The *Pf*aE3 dimer model incorporates an extra anti-parallel β strand in the space that would, in the complex of human E3 and E3BP, be occupied by the E3BP subunit-binding domain (indicated for one chain by a red-dashed ellipse). (**C**) Seven fleximers overlaid to illustrate the scope of conformational space explored by the models generated by the DMD process. Three regions were allowed to flex during the modelling: residues 1–27, 88–123 and 406–420. Key: model 1 (red); model 1000 (orange); model 2000 (light green); model 3000 (dark green); representative fleximer (model 3242, blue); model 4000 (purple); model 5000 (pink). (**D**) The representative fleximer model superimposed on the cluster 2 DAM.

SAXS data (Figure 3A) were acquired on the EMBL beamline X33 at DESY, Hamburg. One day before SAXS, the homogeneity [a single 6.3 S species (results not shown)] of the *Pf*aE3 was confirmed by SV. Two days after SAXS, some of the proteins had degraded resulting in a small amount (5%) of a 3.9 S species in addition to the main 6.3 S species. Protein activity was also confirmed on the day of purification. A maximum at an *sR_g_* just above 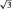 in the dimensionless Kratky plot (*sR*^2^_*g*_*I*(*s*)/*I*(0) versus *sR_g_*) of the SAXS data demonstrated that the protein was folded and elongated and the linear Guinier region in the data at low angles confirmed that there was no aggregation in the sample (results not shown). The radius of gyration, *R_g_*, determined from the Guinier analysis was 37.6±0.3 Å. GNOM [29] was used to determine the distance distribution function, *p*(*r*), from which the maximum dimension of the particle, *D*_max_, was determined (129 Å). The *R_g_* estimated from GNOM analysis, 37.5±0.04 Å, was similar to that determined from the Guinier region. The bell shape of the *p*(*r*) curve (results not shown) has a short tail, indicative of a globular particle which is slightly elongated, in agreement with the dimensionless Kratky analysis. Twenty *ab initio* DAMs (dummy atom models) generated using the program DAMMIF [30] were clustered by DAMCLUST [27] into three groups, cluster 1 (comprising eight models), cluster 2 (comprising five models) and cluster 3 (comprising seven models). The sedimentation coefficient of the representative model of each group was computed using US-SOMO [46,47]. Best agreement was obtained for cluster 2 for which *s*^0^_20, *w*_ was 6.43 S (whereas it was 6.88 and 6.83 S for clusters 1 and 3, respectively) in good agreement with the experimentally determined value (6.2 S).

An atomic resolution model of *Pf*aE3 was generated using the PHYRE2 server [31]. Coordinates were generated for 514 of the 566 residues: the first 12 residues (including the His-tag) were not modelled, and the final residue (also a histidine) was absent. In addition, two longer sequences [of 19 and 20 amino acids (residues 96–114 and 465–484), respectively] were not modelled. They have no structural homologues in the Protein Data Bank [48]. An additional model was generated with the I-TASSER server [32] in which all the residues were modelled. Dimer forms of both models were generated by superimposition on the dimer structure of human E3 (PDB ID: 2F5Z, [33]). Whilst the PHYRE2 monomer superimposed well, residues 406–420 of the I-TASSER monomers sterically clashed. Therefore a composite model was made in which residues 403–418 of the I-TASSER model were replaced with those from the PHYRE2 model. This composite dimer was then superimposed on the human E3 dimer structure without steric clashes. US-SOMO [46,47] was used to compute *s*^0^_20, *w*_ and *R_g_* for this dimer, giving 7.13 S and 31.6 Å, respectively, in poor agreement with the experimentally determined values of 6.2 S and 37.5 Å.

Anticipating that the disagreement between calculated and experimental values might originate from flexibility in extended surface loops observed in the *Pf*aE3 model but not the human structure, 5000 ‘fleximers’ of these loops (residues 88–123 and 406–420) and the first 27 residues (including the His-tag) were generated using the DMD (discrete molecular dynamics; [49,50]) tool in US-SOMO with an Andersen thermostat temperature of 0.5 kcal/mol/kB, where most proteins will not unfold or deviate much from native state (Figure 3C). GAJOE [51] was then used to select an ensemble of fleximers whose combined theoretical scattering intensity gives best agreement with the experimental SAXS data. A representative model is shown in Figure 3(D) for which *s*^0^_20, *w*_ and *R_g_* were computed (using US-SOMO) to be 6.30 S and 33.5 Å, respectively, in better agreement with the experimental data than the initial dimer model. The *Pf*aE3 dimer model incorporates an extra anti-parallel β-strand in the space that would, in the complex of human E3 and E3BP, be occupied by the SBD (sub-unit binding domain) of E3BP (Figure 3B).

The dimer model was also superimposed (using SUPCOMB [52]) on the representative cluster 2 DAM. The overlay highlights a key difference between the two models: the DAM was generated with P2 symmetry constraints, whereas the fleximers were generated from an initially symmetrical dimer in the absence of symmetrical constraints. This observation notwithstanding, the majority of the two models overlay reasonably well (Figure 3D), acknowledging that this model is only one of an ensemble that agrees with experimental data and it is not unreasonable to envisage a slightly different conformer overlaying well with other parts of the DAM not occupied by this model. Thus the SAXS data confirm the dimeric nature of *Pf*aE3 and are consistent with a model in which parasite-specific regions of the protein extend from the globular core.

### Physiological role of *Pf*aE3

**Figure 4:**
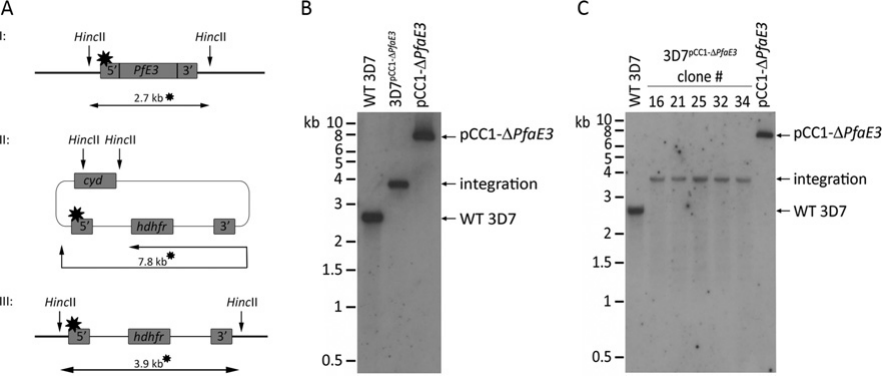
Knockout of the *Pfae3* gene (**A**) A schematic representation (not to scale) of the expected DNA fragment sizes using the 5′ flank of the transfection plasmid (star) following digestion of genomic DNA with HincII. (I) Digestion of the endogenous gene results in a 2.7 kb fragment; (II) digestion of the transfected plasmid pCC1-Δ*PfaE3* results in a 7.8 kb fragment; (III) following integration of the pCC1-Δ*PfaE3* plasmid, digestion of the *ae3* locus with HincII results in a 3.9 kb fragment. (**B**) Southern blot of genomic DNA (2.5 μg) of 3D7 wild-type (WT 3D7), the transfected line *Pf*3D7^∆*PfaE3*^ and the transfected plasmid pCC1-Δ*PfaE3* after digestion with *Hin*cII showed a DNA fragment of 2.7 kb in the 3D7 wild-type genomic DNA diagnostic for the *ae3* gene locus. In the genomic DNA of the transfected parasite line a band diagnostic for the gene deletion (3.9 kb) was detectable and in the lane where the plasmid was loaded, the expected 7.8 kb DNA fragment was detected. (**C**) Southern blot of genomic DNA of *P. falciparum* 3D7 wild-type and five clones (2.5 μg; clones numbered 16, 21, 25, 32 and 34) was digested with HincII and probed as described in (**B**). This confirmed the deletion of the *Pfae3* gene, as only the 3.9 kb integration fragment was detected, in the clonal parasite lines.

**Figure 5:**
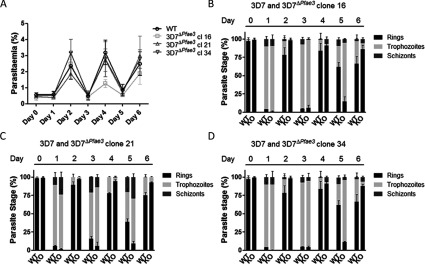
Growth of 3D7 and 3D7^∆*PfaE3*^ Synchronized 3D7 and 3D7^∆*PfaE3*^ clones 16, 21 and 34 were diluted to approximately 0.5% parasitaemia at day 0. The cultures were diluted 1:5 with fresh erythrocytes every 2 days and parasitaemia (**A**) and parasite stage (**B**) were determined in Giemsa stained thin smears over three growth cycles. Data shown represent means of two independent experiments performed in triplicate ± S.D. Abbreviations: WT: 3D7 wild-type; KO: 3D7^Δ*Pfae3*^ clones 16, 21 and 34.

The physiological role of *Pf*aE3 was probed by deleting *ae3* in *P. falciparum* 3D7 (Figure 4). The gene was replaced by the selectable marker *hdhfr* after initial positive selection with WR99210 followed by negative selection using 5-FC (5-fluorocytosine) generating the line 3D7^Δ*ae3*^ (Figure 4B). Several clones were isolated (Figure 4C) and clones 16, 21 and 34 were investigated further. The mutant parasite clones did not show an obvious growth phenotype, although it was noted that they grew extremely synchronous–even after three growth cycles they maintained their synchronicity (Figure 5). These data corroborate that *Pf*aE3 is not essential for parasite survival during intra-erythrocytic growth although their tendency to maintain synchronous growth suggests that the gene deletion affects progression through their cell cycle. Whether this is exerted by affecting the number of merozoites generated per cell or synchronicity of mitotic activity itself or whether it is caused by affecting re-invasion of fresh erythrocytes by daughter merozoites has to be established in future work.

Given the potential role of *Pf*aE3 producing NADH for downstream reductive reactions that may be important for the antioxidant defence of the apicoplast and possibly other compartments of the parasite cell, the susceptibility of inhibitors increasing oxidative stress was determined (Table 2). Reduction of glutathione levels by BSO, a specific inhibitor of γ-glutamylcysteine synthetase [53] had a differential effect on the survival of the parasites, with clones 16 and 34 being more sensitive to inhibition with BSO than clone 21 and wild-type 3D7 (Table 2). This differential effect was even more pronounced when the susceptibility towards paraquat, a known pro-oxidant [54–56] was determined. The 3D7^Δ*ae3*^ clones 16 and 34 showed decreased IC_50_ values (one-third of the IC_50_ value determined for wild-type 3D7), whereas this was not the case for 3D7^Δ*ae3*^ clone 21. These data imply that the deletion of *ae3* results in a differential adaptation of parasites to the gene removal. Triclosan, an inhibitor of FabI (an enoyl-acyl carrier reductase) and a component of fatty acid biosynthesis [57–59], shows differential inhibition of the mutant clones, which appears to be opposite of that found by pro-oxidants.

**Table 2:** Sensitivity to inhibitors The data represent means from two to three independent determinations ±S.D. performed in triplicate.

Inhibitor	IC_50_ 3D7 (μM)	IC_50_3D7^Δ*Pfae3*^ clone 16 (μM)	IC_50_3D7^Δ*Pfae3*^ clone 21 (μM)	IC_50_3D7*^ΔPfae3^* clone 34 (μM)
BSO	32.2±1.0	21.1±1.0	40.4±2.5	21.9±2.0
Paraquat	34.8±4.0	12.0±1.7	37.7±3.1	14.9±1.9
Triclosan	6.2±1.2	13.8±1.7	2.4±0.3	14.3±2.2

**Figure 6:**
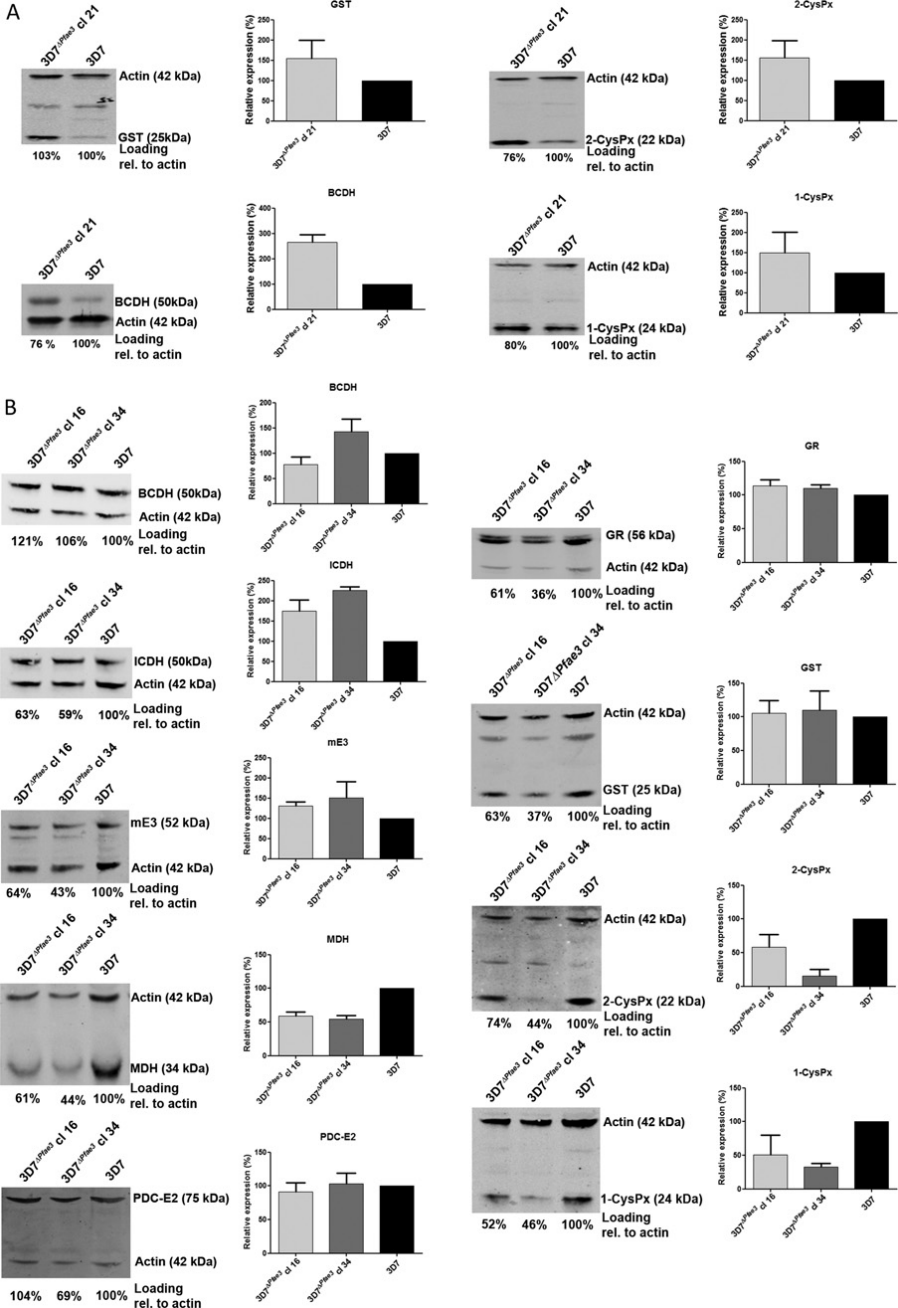
Relative expression levels of proteins involved in redox control (**A**) Parasite lysates of 3D7 and 3D7^Δ*Pfae3*^ clone 21 were generated and 20 μg of protein per lane were separated on SDS–PAGE (4–12% gel), and probed with antibodies raised against a variety of *P. falciparum* proteins and simultaneously with anti-*P. falciparum* actin antibody as a loading control. The secondary antibody used was an IR dye-conjugated antibody, which allowed quantification of signals. The relative expression levels of all proteins were calculated in relation to the actin loading control. The data represent means of three independent blots ± S.D. Antibody sources and dilutions used are given in the Experimental Procedure. (**B**) Parasite lysates of 3D7 and 3D7^Δ*Pfae3*^ clones 16 and 34 were separated on 4–12% SDS–PAGE (4–12% gel), and probed with antibodies raised against a variety of *P. falciparum* proteins with anti-*P. falciparum* actin antibody as a loading control essentially as described in (**A**). The data represent means of three independent blots ± S.D. Antibody sources and dilutions used are given in the Experimental Procedure.

These data prompted us to further assess the physiological state of 3D7^Δ*ae3*^ clones and we analysed potential changes in protein levels involved in the maintenance of the intracellular cytoplasmic redox state (Figure 6). Cytosolic 1-CysPx, PF3D7_0802200) and 2-CysPx, PF3D7_1438900) as well as GST (PF3D7_1419300) were marginally up-regulated in 3D7^Δ*ae3*^ clone 21, suggesting that deletion of *Pfae3* triggers an up-regulation of parasite's antioxidant response (Figure 6A). However, this was not observed in 3D7^Δ*ae3*^ clones 16 and 34, where levels of both peroxiredoxins were lowered and GST was not or only marginally affected (Figure 6B).

The levels of mitochondrial proteins such as branched chain α-keto acid dehydrogenase E2 (BCDH-E2; PF3D7_0303700) were elevated in all mutant parasite clones although this particularly pronounced in clone 21. This tendency correlated well with increased levels of mitochondrial NADP^+^-dependent ICDH (isocitrate dehydrogenase; PF3D7_1345700) as well as slightly elevated levels of mE3 (PF3D7_1232200) in 3D7*^PfΔae3^*clones 16 and 34 (Figure 6B).

The PDC–E2 protein level was unaffected in 3D7*^PfΔae3^*clones 16 and 34 suggesting that this component of PDC is still expressed at normal levels in mutant parasites.

## DISCUSSION

*Plasmodium* possesses a single PDC exclusively found in the apicoplast [15]. The three other KADH complexes are mitochondrial [60] and this distribution requires the presence of apicoplast- and mitochondrion-specific dihydrolipoaminde dehydrogenases (E3) that interact with their organelle-specific KADH [20,61]. In accordance with this distribution, mitochondrial KADH complexes share a single E3 (mE3; PF3D7_1232200), while the apicoplast PDC has its own, apicoplast-located E3 encoded by a separate gene (aE3; PF3D7_0815900) [20]. Experimental evidence suggests that apicoplast-located PDC is not essential for the survival of intra-erythrocytic *Plasmodium yoelii* [16] but that the protein is important for late liver stage development. Recently the gene encoding PDC E1α was also deleted in the human malaria species *P. falciparum* resulting in the arrest of parasite development at the oocyst stage in the *Anopheles* vector [18]. Similar results were obtained when genes encoding components of the type II fatty acid biosynthesis pathway were deleted from *P. falciparum*, supporting the hypothesis that, as in plants, the major role of apicoplast PDC is to provide acetyl-CoA for this biosynthetic pathway [16,19,62].

The potential suitability of PDC as a target for transmission blocking or exo-erythrocytic drug discovery prompted us to recombinantly express *Pfa*E3 and analyse its biochemical properties. Kinetically *Pf*aE3 favours the forward reaction (the oxidation of DHLA and reduction of NAD^+^), rather than the reverse reaction (Table 1). This suggests that in its physiological environment the enzyme is primarily acting as part of apicoplast PDC rather than undergoing redox reactions to assist in maintaining organellar redox and antioxidant homoeostasis, as has been suggested for other organisms [63,64]. The structural stability of *Pf*aE3 was probed by AUC. The whole cell weight average molecular mass at infinite dilution of *Pf*aE3 determined by SE was consistent with that expected for a dimeric species. However during SDS–PAGE *Pfa*E3 migrates as two species, both of which are N-terminally 6-His tagged suggesting that the faster migrating protein may have lost parts of its C-terminus. Deletion of the C-terminal 14 amino acids of *Azotobacter vinelandii* E3 led to its inactivation, decreased its thermostability and also negatively affected FAD-binding to the protein [65]. FAD-binding is required for the correct folding of E3 [66], which, in turn, affects dimer formation. The decrease of molecular size from 64 kDa (computed from the amino acid sequence for 6-His-*Pf*aE3) to the 60–62 kDa observed during SDS–PAGE is consistent with the loss of about 15–34 amino acids and it is possible that this negatively affects *Pf*aE3 co-factor binding and dimerization and thus may modify protein structure and catalytic activity. However, the kinetic parameters of the recombinant protein were comparable with those determined for E3 from other organisms suggesting that only a small proportion of the recombinant protein is negatively affected, which is in agreement with the finding that *Pf*aE3 is stable at high protein concentrations *in vitro*. Overall, the protein expression system used here facilitates the generation of milligram amounts of purified protein that is enzymatically active and appears to be structurally stable, provided it is stored at high concentrations (> 10 μM) and thus provides a suitable system to generate sufficient protein for future drug-screening efforts.

The sedimentation coefficient determined by SV (*s*^0^_20, *w*_ of 6.2±0.1 S) is comparable with that determined for the human E3 dimer (5.9 S, [45]) suggesting that the overall solution conformation might be similar. However, human E3 has a molecular mass of 106 kDa compared with the 128 kDa of *Pf*aE3, so it would not be unexpected for the sedimentation coefficient to be a little higher. The additional loops observed in the *Pf*aE3 model, if real, would serve to somewhat offset the increase in sedimentation coefficient afforded by the additional mass. The dimeric nature of *Pf*aE3 is confirmed by SE and by the SAXS data in which the radius of gyration, maximum particle dimension and DAM all are consistent with a dimer. The positioning of the ‘missing’ loops and N-terminal portions in the proposed model remains undetermined–modelling against one SAXS dataset is inconclusive. This limitation notwithstanding, the AUC and SAXS data are all consistent with a dimeric structure for *Pf*aE3 and are not inconsistent with the augmented homology model.

In addition to the two extra loops, the high-resolution model positions a two-stranded anti-parallel β motif in the space that, in the structure of human E3 plus the SBD of E3BP (PDB ID: 2F5Z, [33]), is occupied by the E3BP SBD. *Pf*aE3 does not interact with E3BP SBD, but rather the SBD of *Pf*E2 since the parasite genome does not encode an *e3bp* homologue. The presence of this ‘extra’ β motif implies that this interaction may be quite different from that observed for human E3-E3BP, supporting the suitability of *Pf*aE3 as a target for the development of intervention strategies.

To further assess *Pf*aE3 function, we generated *Pfae3* null mutants (3D7^Δ*ae3*^). Our study corroborates that the gene/protein is not essential for intra-erythrocytic development in *P. falciparum* 3D7. The deletion of *Pfae3* did not affect parasite growth rates. However, upon careful scrutiny of the intra-erythrocytic development of 3D7^Δ*ae3*^, we observed that the mutant organisms unusually maintained their synchronicity over several growth cycles, while this is not a feature of the wild-type 3D7 line (Figure 5). The causal relationship between this observation and the deletion of *Pfae3* is not clear. A recent study identified *Pf*aE3 to be not only part of PDC but also to be associated with parasite DNA, which possibly suggests a regulatory role for the protein in gene expression, in addition to its function in PDC activity [67]. Another important finding was that the loss of PDC function upon *Pfae3* deletion resulted in an elevation of enzyme levels involved in the generation of acetyl-CoA in the mitochondrion such as BCDH-E2 and mE3. This suggests that the loss of PDC activity may lead to an increased activity of TCA activity in order to compensate for the loss acetyl-CoA provision, which is in agreement with a recent report showing that mitochondrial acetyl-CoA is generated by the branched chain α-keto acid dehydrogenase [68].

In order to test whether the lack of PDC activity impacts on the parasites’ ability to defend themselves against oxidative stress, we exposed three 3D7^Δ*ae3*^ clones to pro-oxidants that challenge their redox homoeostasis. Interestingly, the 3D7^Δ*ae3*^ clones showed a differential response to the inhibitors, which suggests that they adapt in different ways to the loss of *Pf*aE3 function. This is also reflected in our finding that changes in protein levels observed upon *ae3* deletion have clonal specificity (Figure 6). Clones 16 and 34 showed an increased susceptibility to the oxidative stressor paraquat and also (less pronounced) to BSO, which was in agreement with their reduced amounts of 1-CysPx and 2-CysPx, respectively. The susceptibility of clone 21 to these stressors was only marginally reduced and accordingly, the levels of the two antioxidant enzymes were elevated. These data suggest that *Pf*aE3 may play a role in protecting *P. falciparum* against oxidative or xenobiotic challenges [69], although the adaptive responses that the parasites mount show clonal differences and this phenomenon requires further investigation to fully appreciate the flexibility of *Plasmodium* in response to environmental changes.

Overall, the present study has corroborated that *Pf*aE3 is not essential for *P. falciparum* survival during erythrocytic life, but that the protein plays a role independent of apicoplast PDC in maintaining cellular redox homoeostasis and that it impacts on cell cycle progression during this life-cycle stage. These phenotypes require further investigation to fully understand the functions of the protein in addition to being a vital component of apicoplast PDC. In addition, we provide evidence for a dimeric *Pf*aE3 molecule in solution that differs significantly from its human counterpart, thus offering the potential for the development of transmission-blocking or exo-erythrocytic anti-malarial intervention strategies.
